# Pulmonary hemangioma mimicking bronchiectasis: A case report

**DOI:** 10.1097/MD.0000000000011203

**Published:** 2018-06-22

**Authors:** Wei Li, Tong Xin, Yuxin Hu, Peng Gao, Mo Chen, Jie Zhang

**Affiliations:** Department of Respiratory and Critical Care Medicine, the Second Hospital of Jilin University, Changchun, Jilin, China.

**Keywords:** bronchiectasis, enhanced computed tomography, misdiagnosis, pulmonary hemangioma

## Abstract

**Rationale:**

Pulmonary hemangioma is a rare thoracic condition that can lead to hemoptysis. Here we report a case that presented with lumen dilatation suggestive of bronchiectasis on high-resolution computed tomography (CT) and was misdiagnosed as bronchiectasis for more than 10 years.

**Patient concerns:**

A 41-year-old female patient was admitted to the Department of Respiratory Medicine due to hemoptysis.

**Diagnoses:**

The patient was misdiagnosed as having bronchiectasis for more than 10 years. Enhanced chest CT was not performed until treatment of recurrent hemoptysis with antibiotics and hemostatic therapy was no longer effective. With lumen dilation and the “signet ring” sign as the main findings on CT, pulmonary hemangiomas are easily misdiagnosed.

**Interventions:**

A left lower lobe lobectomy was performed, and the postoperative pathology revealed a hemangioma of the left lower lobe of the lung without bronchiectasis.

**Outcome:**

After treatment, the patient no longer had hemoptysis.

**Lessons:**

Therefore, in the clinical diagnosis and treatment of patients presenting with hemoptysis, enhanced CT/CT angiography (CTA) is necessary for differential diagnosis.

## 1 Introduction

Pulmonary hemangiomas are most commonly associated with congenital dysplasia, including pulmonary arteriovenous fistulas and congenital telangiectasia. The condition was first reported by Churton in 1897^[1]^ and is caused by congenital or acquired vascular abnormalities. The main pathological changes leading to the formation of pulmonary hemangiomas are congenital pulmonary vascular ring defects, which induce pulmonary arteriovenous shunting and abnormal blood vessel generation. Acquired pulmonary hemangiomas can occur in cirrhosis, schistosomiasis, and after surgery.^[2]^ Here, we present a case of a pulmonary hemangioma that was initially diagnosed and treated as bronchodilatation for >10 years before the correct diagnosis was made.

## 2 Case Presentation

**Figure 1:**
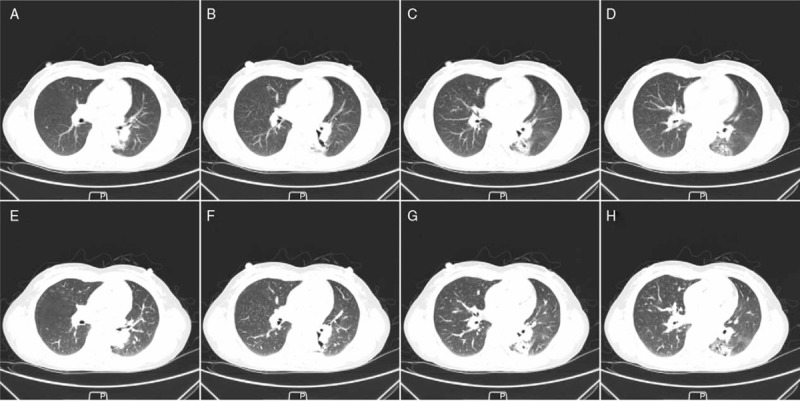
Chest CT images. The area assumed to be bronchiectasis in the lower left lung showed little change over >2 years. A–D, Chest CT images taken in 2014; E–H, chest CT images taken in 2016. CT = computed tomography.

A 41-year-old female patient was admitted to the Department of Respiratory Medicine due to hemoptysis. The patient had first experienced hemoptysis with a small amount of fresh blood and neutral-smelling yellow sputum 10 years previously. She denied breathing difficulties and chest pain. Chest computed tomography (CT) scans met the diagnostic criteria for bronchiectasis^[3]^ combined with infection (Fig. 1). The patient's condition improved after administration of intravenous antibiotics (ceftriaxone and clarithromycin) and intravenous hemostasis (hemocoagulase) treatment. The patient then experienced disease recurrence once or twice per year and was again diagnosed with bronchiectasis combined with infection each time. After treatment with anti-inflammatory and hemostatic therapy, the patient's condition also improved each time. Seven days prior to presentation in our department, the patient began to cough with a small amount of yellow sputum and dark red blood. The same treatment strategy was again given in the local hospital, but the patient's symptoms were not improved. One hour prior to presentation in our department, the patient's symptoms became aggravated with increased hemoptysis of about 100 mL fresh blood combined with chest pain and difficulty breathing.

**Figure 2:**
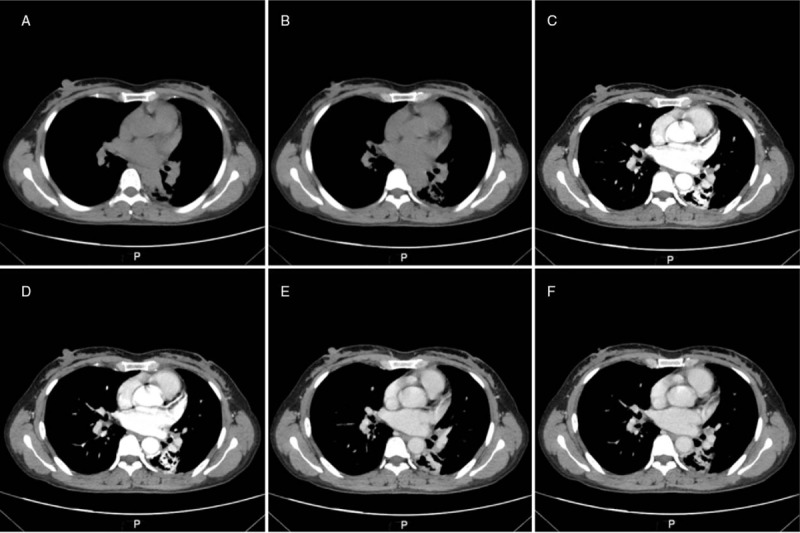
Enhanced chest CT images revealed (A–B) an enlarged lumen of the left dorsal bronchus, which was larger than the accompanying pulmonary artery as well as thick, straight, and uniform walls of the bronchi with the “signet ring” appearance; and (C–F) an enhanced “bronchi wall” in the arterial phase with decreased intensity in the venous phase.

Laboratory tests showed a white blood cell count of 13.5 × 10^9^/L, neutrophil percentage of 76%, lymphocyte percentage of 17%, neutrophil count of 10.33 × 10^9^/L, and monocyte count of 0.8 × 10^9^/L. The test for tuberculin was negative. Blood gas analysis revealed an oxygen concentration of 33%, pH of 7.36, PCO_2_ of 44 mmHg, PO_2_ of 60 mmHg, HCO_3_^−^ of 24.9 mmol/L, and base excess of –0.8 mmol/L. Tests of coagulation time, liver function, and kidney function showed no abnormalities. Chest CT showed increased patchy high-density shadows in the left lower lobe of the lung with visible bronchial broadening as changes to the patient's “bronchiectasis” (Fig. 2).

**Figure 3:**
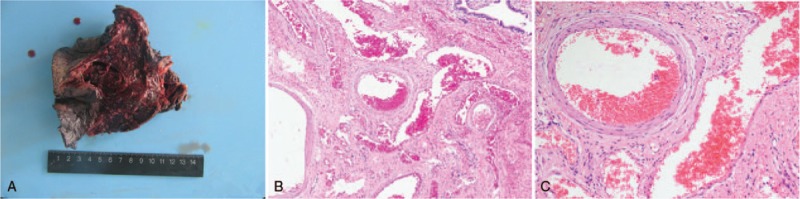
Images of excised lung tissue and pathology sections. The morphology of the resected artery wall was consistent with a pulmonary hemangioma with mainly thick-walled vessels. There was no obvious manifestation of bronchiectasis.

The patient received a 7-day course of intravenous antibiotics (moxifloxacin) and hemostasis therapy (hemocoagulase/ethylenediamine diaceturate injection) as empiric therapy after admission. The patient's symptoms improved with reductions in coughing, sputum discharge, and hemoptysis involving mostly old clotted blood only. Then, enhanced chest CT showed an increased texture of the 2 lungs, widening of the bronchus of the lower portion of the left lower lobe, thickening of the adjacent left lower pulmonary veins and arteries, enhancement of the arterial phase of enhancement, reduction of venous phase enhancement, an increase in the patchy blur density around the trachea, and the trachea. The opening of the main bronchus was unobstructed. Lymph nodes were not seen in the mediastinum, and no obvious effusion was seen in the chest. A left lower lobe lobectomy was performed, and the postoperative pathology revealed a hemangioma of the left lower lobe of the lung without bronchiectasis (Fig. 3). Three months later, the patient had no hemoptysis, sputum, or dyspnea. Lung CT showed only the expected postoperative change and no abnormal shadows or bronchiectasis.

## 3 Discussion

Hemoptysis is the expectoration of blood from the lower respiratory tract and may be life threatening. Thus, hemoptysis is a serious complaint in respiratory medicine. The bleeding can originate from either small or large vessels within the lung.^[4]^ Hemoptysis has multiple causes commonly classified as parenchymal diseases, airway diseases, and vascular diseases. Specifically, the conditions that most commonly cause hemoptysis are bronchiectasis, tuberculosis, fungal infections, cancer, and pulmonary artery aneurysm.^[5,6]^ Various approaches for investigating the cause of hemoptysis as well as various management protocols have been proposed, and researchers worldwide have advocated for a standardized approach to treating patients who present with hemoptysis. Obviously, the cause influences the clinical outcome and prognosis of patients.

In the present case, the patient complained of chronic cough, recurrent infection, and hemoptysis. Chest CT showed an enlarged left dorsal bronchus lumen, which was greater than its accompanying pulmonary artery. The bronchi had thick, straight, and uniform walls with a “signet ring” appearance. Therefore, the patient was diagnosed with and treated for bronchiectasis for >10 years. The patient experienced regular recurrence, but antibiotics and hemostatic drugs had a positive effect each time. Ultimately, however, intravenous contrast CT examination demonstrated the “bronchi wall” was obviously enhanced in the arterial phase and significantly weakened in the venous phase. Therefore, a diagnosis of hemangioma caused by a pulmonary artery aneurysm was made through intravenous contrast CT, and surgical treatment confirmed this diagnosis.

Pulmonary hemangioma is an extremely rare form of benign tumor in the lung.^[5,6]^ It can occur following detachment of vascular cells or be caused by a defect in vascular network development during the embryonic period. Each case can lead to local hyperplasia and endothelial cell cord formation, and numerous hemangiomas can form as blood vessel cells continue to differentiate. Small lesions in early stage tumors may have no clinical symptoms, and thus, are usually difficult to find. After some degree of continued growth, the tumor can induce intermittent cough, chest tightness, chest pain, dizziness, palpitations, hemoptysis, and other symptoms. Moreover, advanced hemoptysis can occur, with rupture of the thoracic hemorrhage. Selective pulmonary angiography can display all the relevant vessels and surrounding hemangiomas, and is thus the only reliable method for diagnosis of a pulmonary hemangioma.

The imaging findings for pulmonary hemangiomas usually reveal a single lesion, but multiple hemangiomas are possible. Pulmonary nodules are mostly distributed in the peripheral field, but some may present with air crescent sign on imaging^[7]^ or as a large solitary pulmonary mass.^[8]^ Chest CT, especially with enhancement or CT angiography, can clearly detect the drainage artery and vein around these nodules through an obvious contrast enhancement effect. Typically, hemangiomas present on CT scans as a delineated mass with good enhancement.^[9]^ High-, iso-, and low-attenuating areas indicate angiomatous, sclerosing, and cystic components, respectively, and each presentation is possible. One study reported that calcification was observed in 41% of hemangioma patients.^[10]^ Small tumors <3 cm in diameter usually show strong homogeneous enhancement, whereas larger tumors may exhibit inhomogeneous enhancement, with cystic components causing poor enhancement in some areas.^[11]^ Other imaging features have been reported, including a tail sign formed by a tail-like projection from the tumor, an air-meniscus sign similar to that seen for aspergilloma, and a prominent pulmonary artery caused by the increased arterial demand of the tumor.^[12]^

Surgical excision of hemangiomas is curative with no need for additional treatment in most cases. For large hemangiomas, lymph node dissection may be appropriate due to the potential for lymph node metastasis. In the present case, the hemangioma seemed to be benign, as no change in its appearance on CT imaging was observed over 10 years. The patient did experience recurrent hemoptysis though, which ultimately led to the correct diagnosis and surgical excision. Although a small asymptomatic tumor can be followed up, a larger tumor with obvious symptoms should be surgically resected as soon as possible given that spontaneous rupture of the hemangioma could cause fatal bleeding.

Taken together, in the clinic, sometimes diagnosis of pulmonary hemangioma cannot be made early, especially when a pulmonary hemangioma shows none of the typical features on CT imaging, just as in the case report. Moreover, patients with pulmonary hemangioma often experience a long history and obvious manifestation, including cough, sputum, and hymoptysis, which are also common in bronchiectasis.^[5,6]^ In addition, imaging of pulmonary hemangiomas usually shows pulmonary nodules,^[8]^ and the “signet ring appearance” is not common. In this case, enhanced contrast CT of the chest is very important for differentiating pulmonary hemangioma and bronchiectasis.

## 4 Conclusion

In summary, pulmonary hemangiomas are rarely encountered in the clinic, and small pulmonary hemangioma lesions have no specific manifestations on CT imaging, which increases the chance for misdiagnosis. Recurrent hemoptysis is the main complication of pulmonary hemangiomas, and intravenous contrast CT is necessary to clarify the diagnosis. The patient in the present case had been misdiagnosed with bronchiectasis for >10 years, because the hemangioma mimicked bronchiectasis on CT scans. This case illustrates that intravenous contrast CT should be performed upon the first presentation of hemoptysis with any abnormality on non-contrast CT imaging, even when bronchiectasis is suspected.

## Author contributions

LW, TX, and YH carried out the data collection, literature review, and drafting of the manuscript. CM contributed to the drafting of the manuscript and aided in the literature review. JZ participated in the data collection and the drafting of the manuscript. PG helps to draft the manuscript and revised the final version of the manuscript. All authors read and approved the final manuscript.

**Data curation:** Tong Xin.

**Investigation:** Mo Chen.

**Software:** Yu Xin Hu.

**Supervision:** Peng Gao.

**Writing – original draft:** Wei Li.

**Writing – review and editing:** Jie Zhang.
